# Phenotyping of autoreactive B cells with labeled nucleosomes in 56R transgenic mice

**DOI:** 10.1038/s41598-017-13422-z

**Published:** 2017-10-16

**Authors:** Vincent Gies, Delphine Bouis, Mickaël Martin, Jean-L. Pasquali, Thierry Martin, Anne-S. Korganow, Pauline Soulas-Sprauel

**Affiliations:** 1 CNRS UPR 3572 “Immunopathology and Therapeutic Chemistry”/Laboratory of Excellence Médalis, Institute of Molecular and Cellular Biology (IBMC), Strasbourg, France; 2 Department of Clinical Immunology and Internal Medicine, National Reference Center for Autoimmune Diseases, Hôpitaux Universitaires de Strasbourg, Strasbourg, France; 3 UFR Médecine, Université de Strasbourg, Strasbourg, France; 4 UFR Sciences pharmaceutiques, Université de Strasbourg, Illkirch-Graffenstaden, France

## Abstract

The phenotypic characterization of self-reactive B cells producing autoantibodies is one of the challenges to get further insight in the physiopathology of autoimmune diseases. We took advantage of our previously developed flow cytometry method, using labeled nucleosomes, prominent autoantigens in systemic lupus erythematosus, to analyze the phenotype of self-reactive B cells in the anti-DNA B6.56R mouse model. We showed that splenic anti-nucleosome B cells express mostly kappa light chains and harbor a marginal zone phenotype. Moreover, these autoreactive B cells fail to acquire a germinal center phenotype and are less abundant in the transitional T3 compartment. In conclusion, the direct detection of autoreactive B cells helped determine their phenotypic characteristics and provided a more direct insight into the B cell tolerance process in B6.56R mice. This method constitutes an interesting new tool to study the mechanisms of B cell tolerance breakdown in B6.56R mice crossed with autoimmune prone models.

## Introduction

B cells play an important role in the development and pathogenesis of autoantibody (autoAb) mediated autoimmune diseases. In healthy individuals, after a stochastic rearrangement of B cell receptors (BCR) during VDJ recombination, up to 75% of newly generated immature B cells of the bone marrow (BM) are autoreactive^1^. But the relatively low occurrence of autoimmune diseases, i.e. 3–8%^2^, implies that mechanisms exist to remove these autoreactive B cells or to render them unresponsive. Maintenance of B cell tolerance occurs at various checkpoints during B cell development. Central B cell tolerance mechanisms take place within the BM, and include clonal deletion, anergy, and receptor editing. B cells that leave the BM express a functional BCR and migrate to the peripheral lymphoid organs to mature^3–7^. However, some autoreactive B cells still reach the periphery. Therefore, additional peripheral tolerance checkpoints exist to remove these pathogenic B cells and include notably clonal deletion, anergy, and inhibition by regulatory T or B cells^3–9^.

Failure in one or more of these mechanisms may lead to tolerance breakdown and development of autoimmune diseases, such as systemic lupus erythematosus (SLE)^10^. This progressive and complex disease is notably characterized by a defect in apoptotic cell clearance, leading to autoAb production against various nuclear antigens, especially double-strand DNA (dsDNA) and nucleosomes. This results in immune complex deposits in blood vessels and different organs, responsible for both systemic and local chronic inflammation^11–16^. Tolerance breakdown is an early event as anti-nucleosome autoAbs can be detected up to 10 years prior the first symptoms of the disease^17,18^. One of the remaining open questions is the precise phenotype of the autoreactive B cells producing these pathogenic antinuclear autoAbs. Most of our knowledge comes from the analysis of B cell hybridomas generated with B cells from anti-DNA transgenic mice^19^. Although these studies provided primordial data regarding tolerance mechanisms, the technology used is not suitable for large scale analysis and does not allow a direct phenotypic approach^20,21^. We previously developed a novel flow cytometry-based method to identify self-reactive B cells with fluorescent nucleosomes in B6.56R mice^22^. Nucleosomes constitute the main autoantigen in SLE. Indeed, although anti-dsDNA Abs are the most common autoAbs observed in SLE^12^, free DNA is usually rare and rather exist in the form of circulating nucleosomes, suggesting that nucleosomes constitute both the driving immunogens and the targets of anti-dsDNA antibodies^18,23^. Moreover, nucleosomes have multiple autoepitopes^24,25^, allowing the detection of a large spectrum of representative pathogenic B cells. The 56R anti-dsDNA heavy (H) chain knock-in mouse model is a useful tool to study B tolerance towards ubiquitous autoantigens, such as nucleosomes^26^. The 56R transgene, a mutated form of the anti-3H9 DNA H chain, forms a BCR with an anti-DNA specificity when combined with almost all endogenous light chains. On a C57BL/6 (B6) background, the 56R mutation (B6.56R) leads to a partial loss of tolerance^19,27^, allowing autoAb production with high affinity for dsDNA and nuclear components^19,26,28^. The B6.56R mouse has an in-frame 56R H chain rearrangement (constant region of “a” haplotype) on one allele (i.e. the transgenic allele), and normal B6H chain genes (constant region of “b” haplotype) on the other. In this model, detection of autoreactive B cells is based on the identification of cells carrying the transgene by PCR, or by flow cytometry using anti-haplotype antibodies. Indeed, the two H chain alleles can be differentiated using anti-IgM^a^ antibodies that specifically bind to IgM with an heavy chain constant region of “a” haplotype (corresponding to the transgenic H chain), and anti-IgM^b^ antibodies that bind to BCR expressed by the endogenous allele (“b” haplotype)^19^. However, editing of variable regions or pairing with specific endogenous light chains (e.g. Vκ21) are known to abrogate DNA binding of the 56R H chain^19,26^ and only 36% of the spontaneous hybridomas produced from B6.56R B cells recognize DNA^26^. Therefore, identification of the 56R H chain with only anti-IgM^a^ labeling is not ideal to analyze autoreactive B cells in this model. For these reasons, we used labeled nucleosomes to characterize B cells based on their genuine autoreactivity^22^. In addition, B6.56R mice do not develop illness despite production of anti-dsDNA autoAbs^26^, unless they are crossed with autoimmune prone mice^29,30^. Thus, our method could help decipher how autoreactive B cells are tolerized in this model.

In this report, we directly phenotyped splenic anti-nucleosome B cells in B6.56R mice and showed that most of them are kappa (κ) positive, harbor a marginal zone (MZ) phenotype with only few with follicular (FO) B cells characteristics. In addition, they failed to acquire a germinal center (GC) phenotype. These results reflect a means to avoid any pathogenic activation of these anti-nucleosome B cells. Indeed autoreactive B cells are likely promoted into locations where their chances of inappropriate activation decrease.

## Results

### Splenic B cell subsets modifications in B6.56R mice

**Figure 1:**
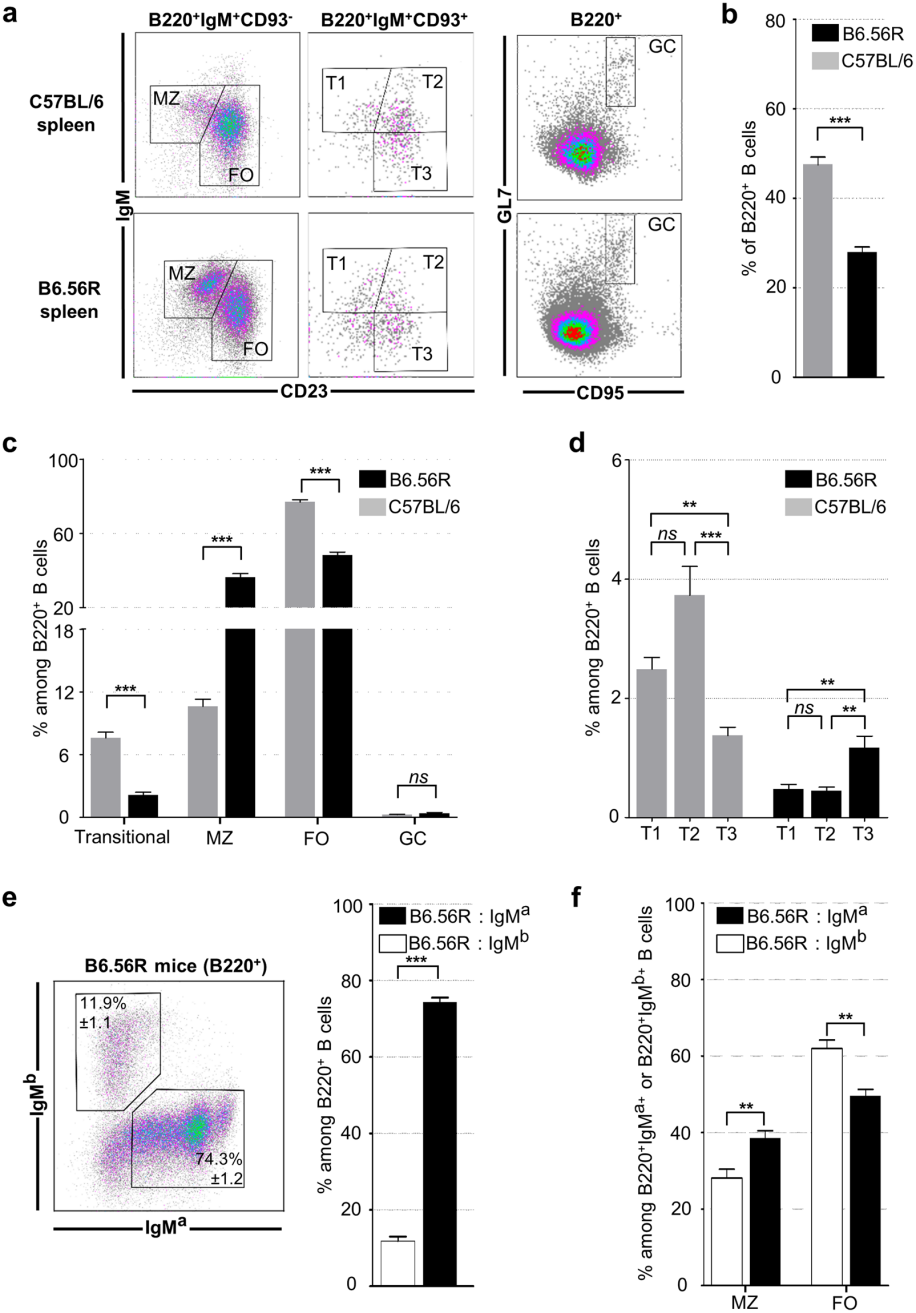
B6.56R mice display lower proportion of total B cells, transitional B cells and FO B cells but a higher MZ B cell frequency, compared to C57BL/6 mice. (**a**) Gating strategy of the different splenic B cell subsets. (**b**) B220^+^ B cells frequency in B6.56R mice *(black)* compared to C57BL/6 control mice *(grey)*. (**c**) Frequency of the different splenic B cell subsets (percentage among B220^+^ cells) in B6.56R mice *(black)* compared to C57BL/6 mice (grey). (**d**) Transitional B cell frequency (percentage of B220^+^ cells) in B6.56R mice *(black)* and C57BL/6 mice *(grey)*. **(e)** IgM^a+^ and IgM^b+^ B cell frequency (percentage of B220^+^ cells) in B6.56R mice. **(f)** Frequency of MZ or FO B cells, among IgM^a+^ *(black)* or IgM^b+^ *(white)* B cell populations in B6.56R (indicated as the percentage of cells among IgM^b+^B220^+^ or IgM^a+^B220^+^ cells). n ≥ 7 in each group; mean ± SEM; spleen; mice aged from 2 to 8 months old. Two-tailed Mann-Whitney U-test. *P < 0.05, **P < 0.005, ***P < 0.001.

Based on the gating strategy defined in Fig. 1a, the different splenic B cell subsets were evaluated by flow cytometry. B220^+^ B cells were decreased both in absolute number and frequency in B6.56R mice compared to C57BL/6 control mice (12.4*10^6^ ± 2.1 *vs*. 34.4*10^6^ ± 5.3 and 27.9% ± 1.2 *vs*. 47.5% ± 1.7 respectively) (Fig. 1b and Supplementary Table 1). MZ B cells were increased in transgenic mice (4.8*10^6^ ± 0.6 in B6.56 R *vs*. 2.9*10^6^ ± 0.4 in C57BL/6 and 36.2% ± 2.0 *vs*. 10.6% ± 0.7%) while FO B cells were decreased both in absolute and relative numbers (8.1*10^6^ ± 2.4 *vs*. 23.8*10^6^ ± 3.2 and 48.2% ± 1.6 *vs*. 76.8% ± 1.3). Among the more immature B cell subsets, transitional B cells were decreased in B6.56R compared to C57BL/6 mice (0.5*10^6^ ± 0.1 *vs*. 2.1*10^6^ ± 0.2 and 2.1% ± 0.3 *vs*. 7.6% ± 0.5 respectively) (Fig. 1c and Supplementary Table 1). Further analysis showed a majority of T2 B cells in controls (3.7% ± 0.5 of B220^+^ cells) while the T3 compartment was the main transitional subset in transgenic mice (1.2% ± 0.2 of B220^+^ cells) (Fig. 1d). These data are in accordance with the literature^26^. In addition, no significant difference was found in the GC subset, neither in relative nor in absolute numbers (Fig. 1c and Supplementary Table 1).

We analyzed the heavy chain usage in B6.56R mice, in order to determine if the increase of MZ B cells is related to a potential sequestration of B cells with the 56R transgenic heavy chain (stained with anti-IgM^a^ antibody). The majority of splenic B220^+^ B cells were IgM^a+^ (74.3% ± 1.2) (Fig. 1e). These cells preferentially harbor a MZ phenotype compared to IgM^b+^ (38.6% ± 1.1 *vs*. 28.1% ± 2.4), whereas there was a higher frequency of IgM^b+^ in the FO B cell compartment (62% ± 2.7 *vs*. 49.6% ± 1.8) (Fig. 1f). These data are consistent with a preferential migration of potential autoreactive B cells into the MZ. However, only a fraction of IgM^a+^ B cells have a self-reactive BCR, due to receptor editing of variable regions or pairing of transgenic heavy chain with specific endogenous light chains^19,26^. Thus, we decided to further analyse the phenotype of autoreactive B cells using a flow cytometry based-assay using labeled nucleosomes^22^.

### Phenotype of anti-nucleosome B cells

**Figure 2:**
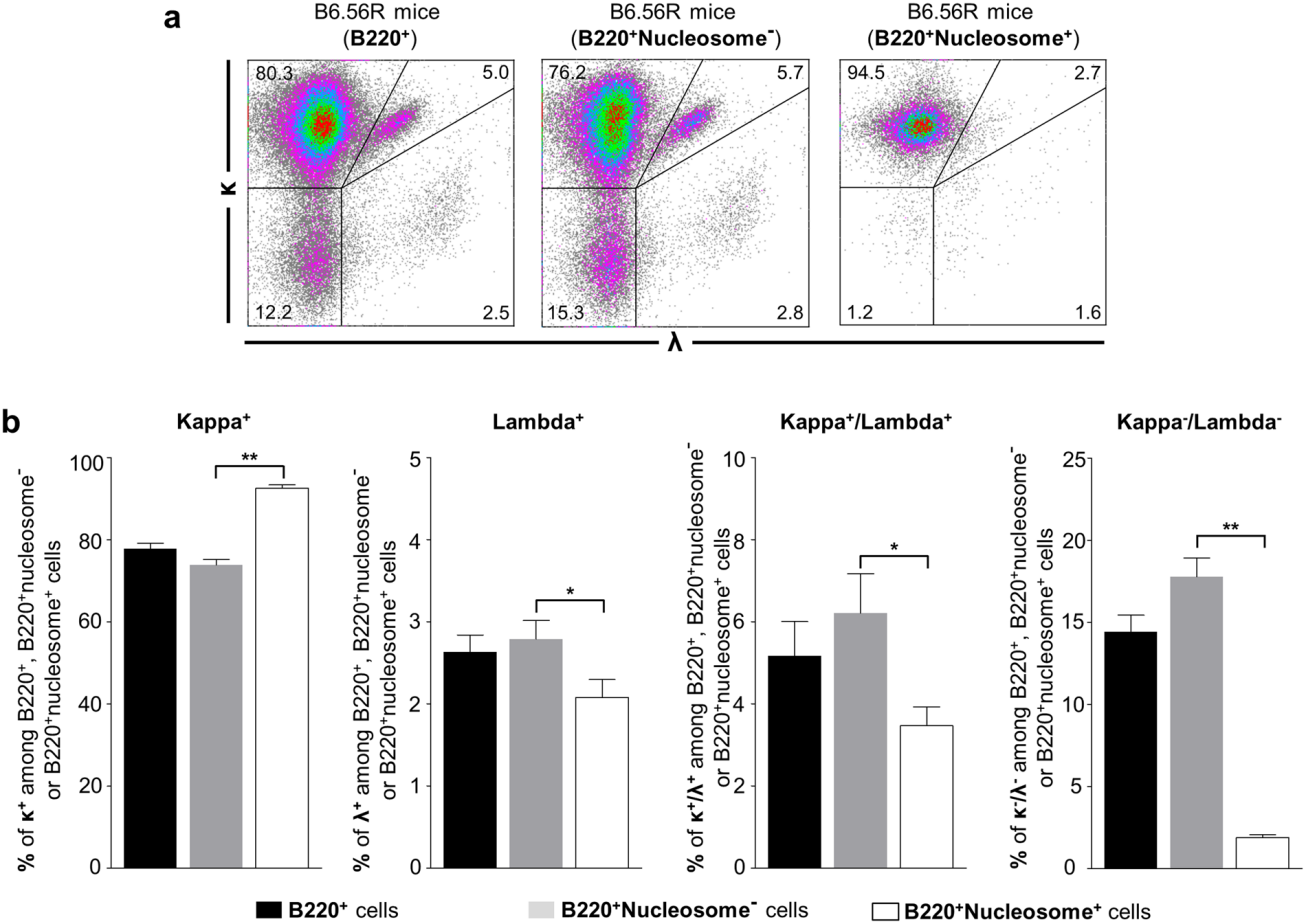
In B6.56R mice, nucleosome^+^ B cells mainly express the κ light chain. (**a**) Representative plots of κ and λ staining among B220^+^, B220^+^nucleosome^−^ or B220^+^nucleosome^+^ cells from B6.56R mice. (**b**) Frequency of κ^+^, λ^+^, κ^+^/λ^+^ or κ^−^/λ^−^ B cells (indicated as percentage among B220^+^, B220^+^nucleosome^−^ or B220^+^nucleosome^+^ cells) in B6.56R mice. n = 5; spleen; mean ± SEM; 4 months old mice. Two-tailed Mann-Whitney U-test. *P < 0.05, **P < 0.005.

In accordance with our previous results^22^, a significant fraction of B cells from B6.56R mice were labeled with nucleosomes. This staining was almost specific to B cells expressing the 56R heavy chain, and only few B cells from C57BL/6 were labeled (Supplementary Fig. 1). First, we assessed the light chain usage in B6.56R mice (Fig. 2). Considering all B220^+^ B cells, 77.8% ± 1.4 expressed κ chains, 2.6% ± 0.2 lambda (λ) chains and 5.2% ± 0.8 co-expressed both chains. In a fraction of B cells (14.4% ± 1.0), no light chain expression could be detected using commercially antibodies against κ or λ (Fig. 2 and Supplementary Table 2). This latter population could be putative Vλx expressing B cells, a light chain that abrogates DNA-binding^31,32^. Further analysis showed a higher frequency of κ expressing B cells in nucleosome^+^ compared to nucleosome^−^ B cells compartment (92.6% ± 0.8 *vs*. 73.9% ± 1.3 respectively), with a low frequency of both λ^+^ B cells (2.1% ± 0.2 *vs*. 2.8% ± 0.2) and B cells expressing dual receptors (3.5% ± 0.5 *vs*. 6.2% ± 1.0). Finally, the proportion of cells without any κ or λ detection was almost negligible among the nucleosome^+^ B cells (Fig. 2b and Supplementary Table 2).

**Figure 3:**
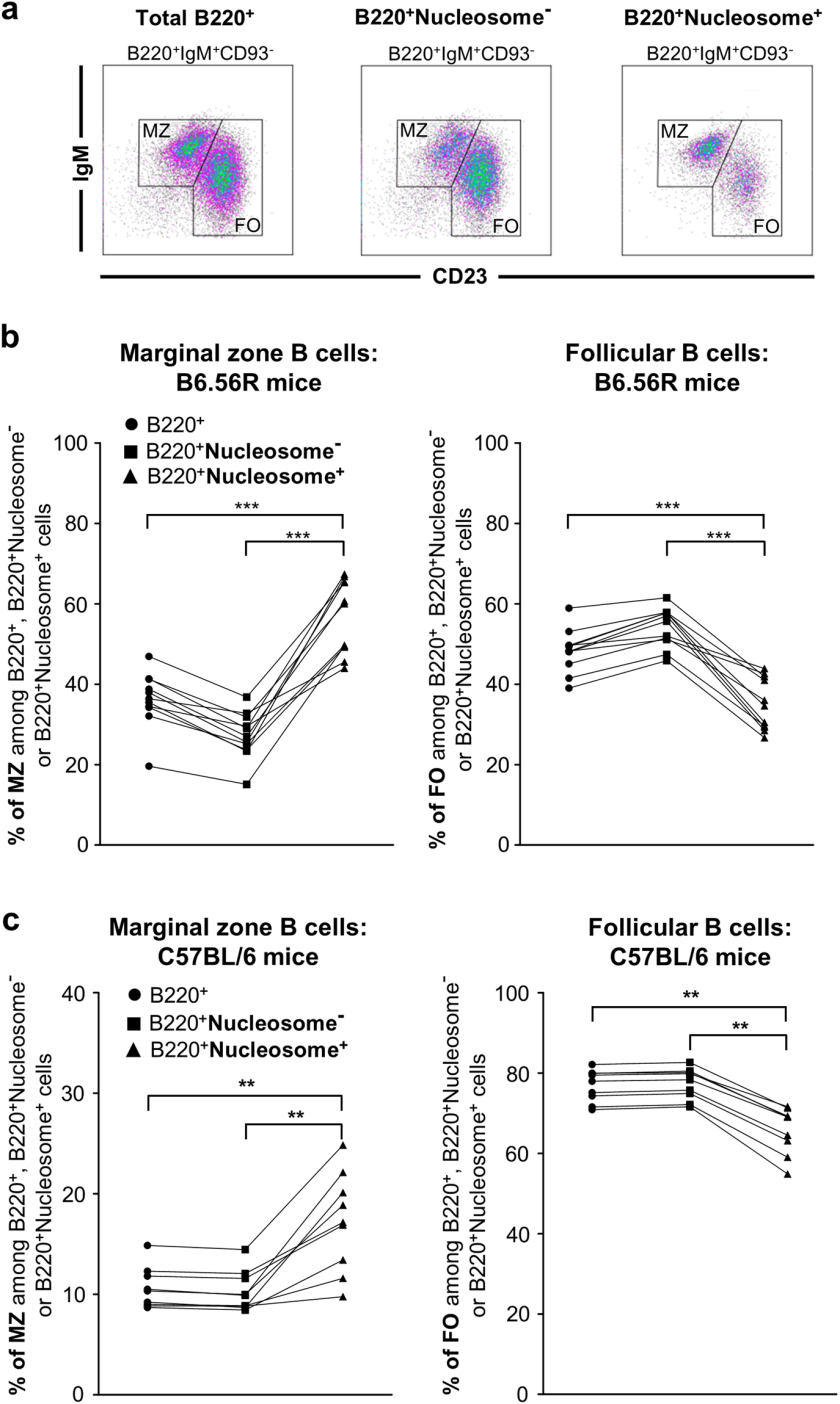
In B6.56R mice, nucleosome^+^ B cells harbor a MZ phenotype. (**a**) Gating strategy for flow cytometry analysis of total B220^+^ cells (left), nucleosome^−^ B cells (middle) and nucleosome^+^ B cells (right) in B6.56R mice. (**b**,**c**) Frequency of total B cells (●), nucleosome^−^ (■) and nucleosome^+^ B cells (▲) in MZ and FO B cell compartments in B6.56R (**b**) and C57BL/6 (**c**) mice (indicated as percentages among B220^+^, B220^+^nucleosome^−^ or B220^+^ nucleosome^+^ cells). n ≥ 9 in each group; spleen; mice aged from 2 to 8 months old. Two-tailed Wilcoxon matched paired signed-rank test. *P < 0.05, **P < 0.005, ***P < 0.001.

**Figure 4:**
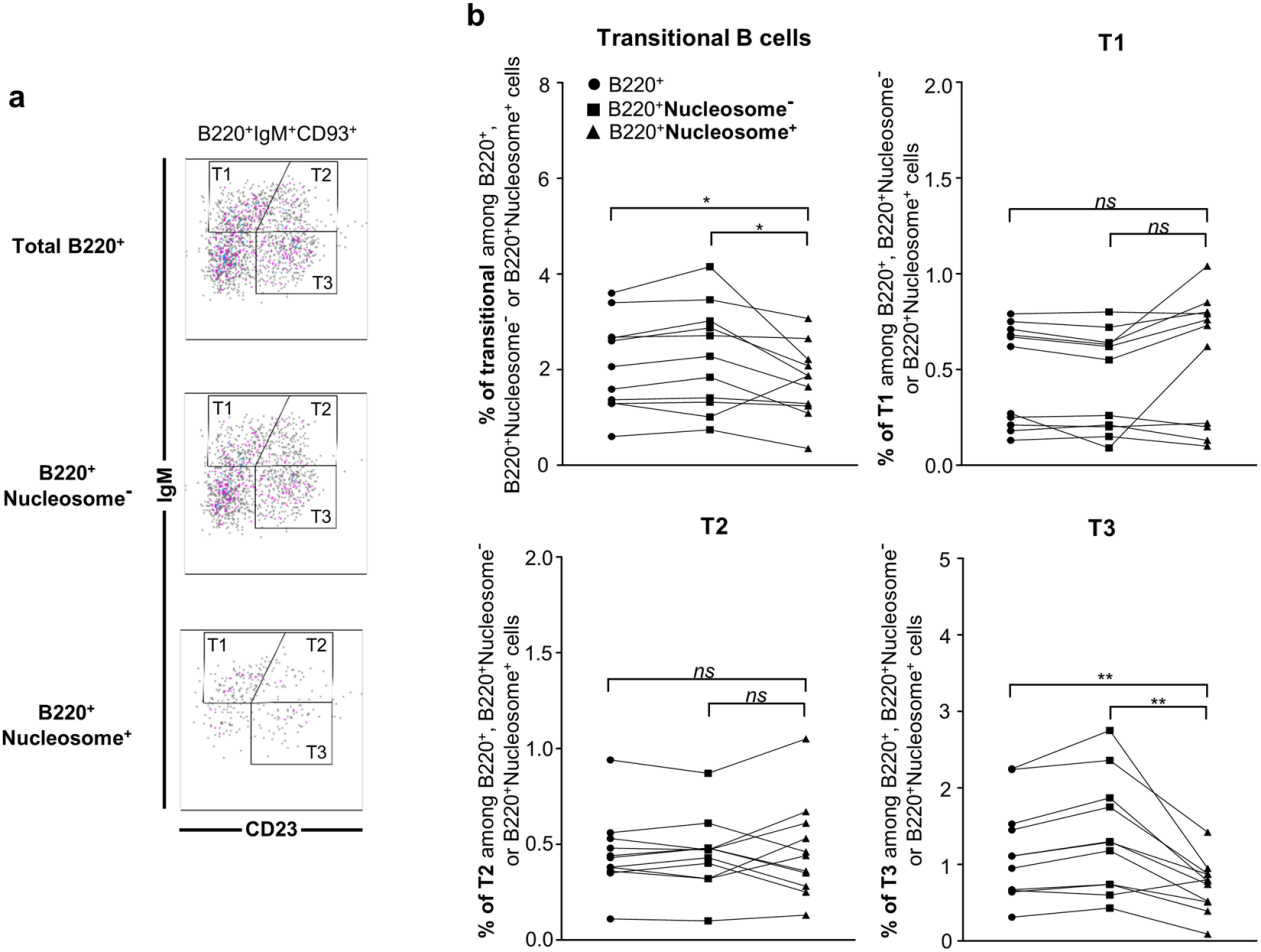
B6.56R mice show decreased frequency of nucleosome^+^ B cells in the transitional B cell compartment, with a specific lower representation of T3 B cells. (**a**) Gating strategy for flow cytometry analysis of total B220^+^ cells (left), nucleosome^−^ B cells (middle) and nucleosome^+^ B cells (right) in B6.56R mice. (**b**) Frequency of total B cells (●), nucleosome^−^ (■) and nucleosome^+^ B cells (▲) in total, T1, T2 and T3 transitional B cell compartments in B6.56R mice (indicated as percentages among B220^+^, B220^+^nucleosome^−^ or B220^+^ nucleosome^+^ cells). n = 11; spleen; mice aged from 2 to 8 months old. Two-tailed Wilcoxon matched paired signed-rank test. *P < 0.05, **P < 0.005, ***P < 0.001.

**Figure 5:**
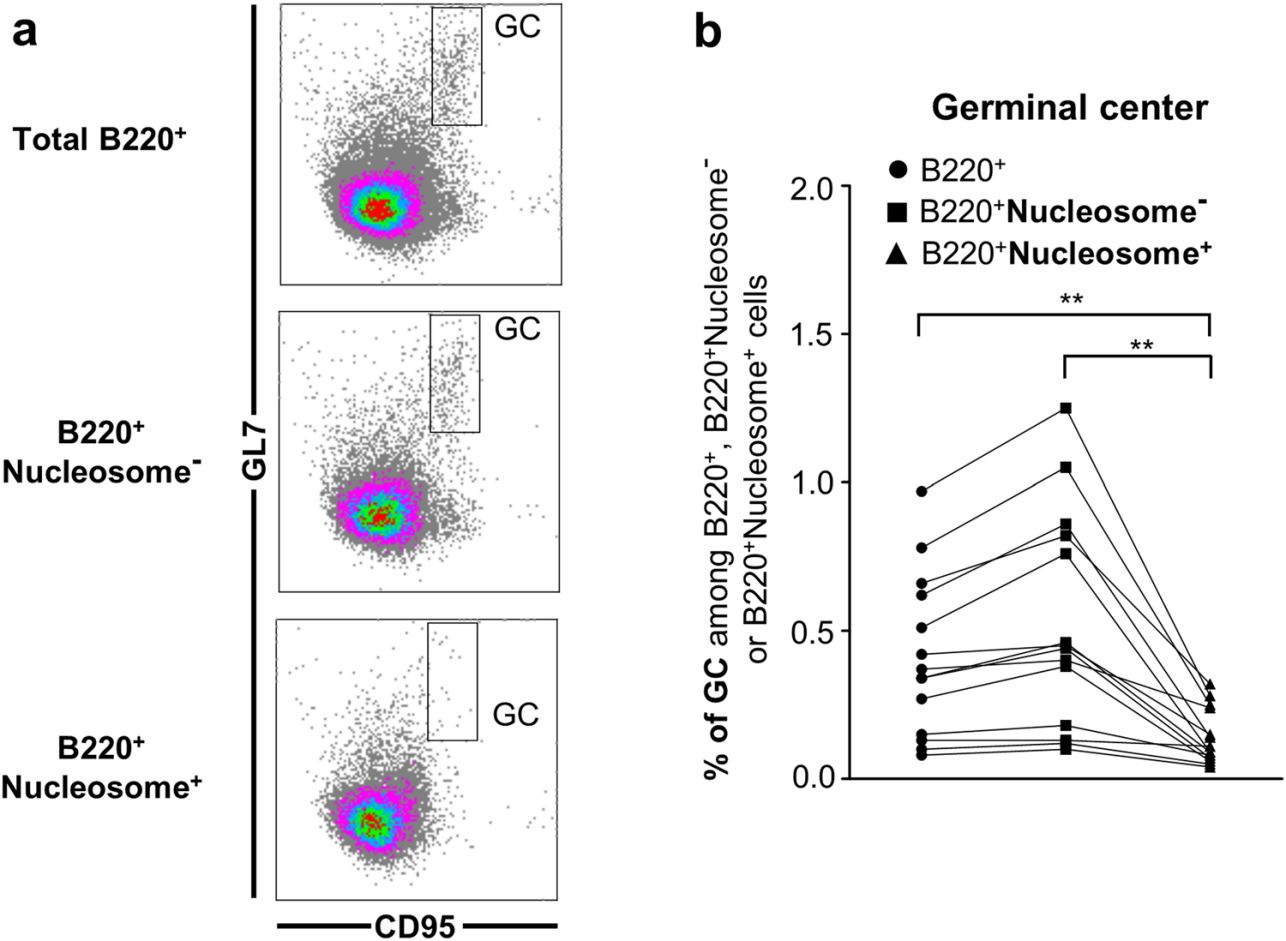
In B6.56R mice, nucleosome^+^ B cells fail to acquire a germinal center phenotype. (**a**) Gating strategy for GC B220^+^ cell, nucleosome^−^ and nucleosome^+^ B cell analysis (left). (**b**) Frequency of total B cells (●), nucleosome^−^ (■) and nucleosome^+^ B cells (▲) in GC in B6.56R mice (indicated as percentage among B220^+^, B220^+^nucleosome^−^ or B220^+^nucleosome^+^ cells (right). n = 14; spleen; mice aged from 2 to 8 months old. Two-tailed Wilcoxon matched paired signed-rank test. *P < 0.05, **P < 0.005.

Compared to nucleosome^−^ B cells, those that bound nucleosomes were chiefly associated with a MZ phenotype (56.7% ± 2.7 nucleosome^+^ *vs*. 27.2% ± 1.7 nucleosome^−^) and a low representation of FO B cells (35.0% ± 1.9 nucleosome^+^ *vs*. 54.1% ± 1.5 nucleosome^−^). These data argue for FO exclusion and MZ sequestration of nucleosome^+^ B cells (Fig. 3a,b and Supplementary Table 2). Interestingly, although there were very few nucleosome^+^ B cells in C57BL/6 mice, these cells harbor a similar phenotype compared to B6.56R mice, with an increased frequency in the MZ compartment at the expense of the FO compartment compared to nucleosome^−^ B cells (17.2% ± 1.6 nucleosome^+^ *vs*. 10.3% ± 0.7 nucleosome^−^ MZ B cells and 65.8% ± 1.9 nucleosome^+^ *vs*. 77.3% ± 1.3 nucleosome^−^ FO B cells respectively) (Fig. 3c). These results suggest the existence of a potential similar MZ sequestration mechanism in wild-type mice. With regard to the more immature B cells in B6.56R mice, a lower frequency of total transitional B cells was found in the nucleosome^+^ compared to the nucleosome^−^ B cells (1.8% ± 0.2 *vs*. 2.3% ± 0.3 respectively), with a lower proportion of T3 subset (Fig. 4 and Supplementary Table 2). Finally, few GC B cells were found among nucleosome^+^ B cells (0.14% ± 0.02 *vs*. 0.58% ± 0.09), indicative of a potential GC exclusion of autoreactive B cells (Fig. 5 and Supplementary Table 2). Similar results were obtained when only the IgM^a+^ B cell population was analyzed (Supplementary Fig. 2), supporting the conclusion that these changes are directly related to self-reactivity and are not solely the consequence of 56R transgene expression.

**Figure 6:**
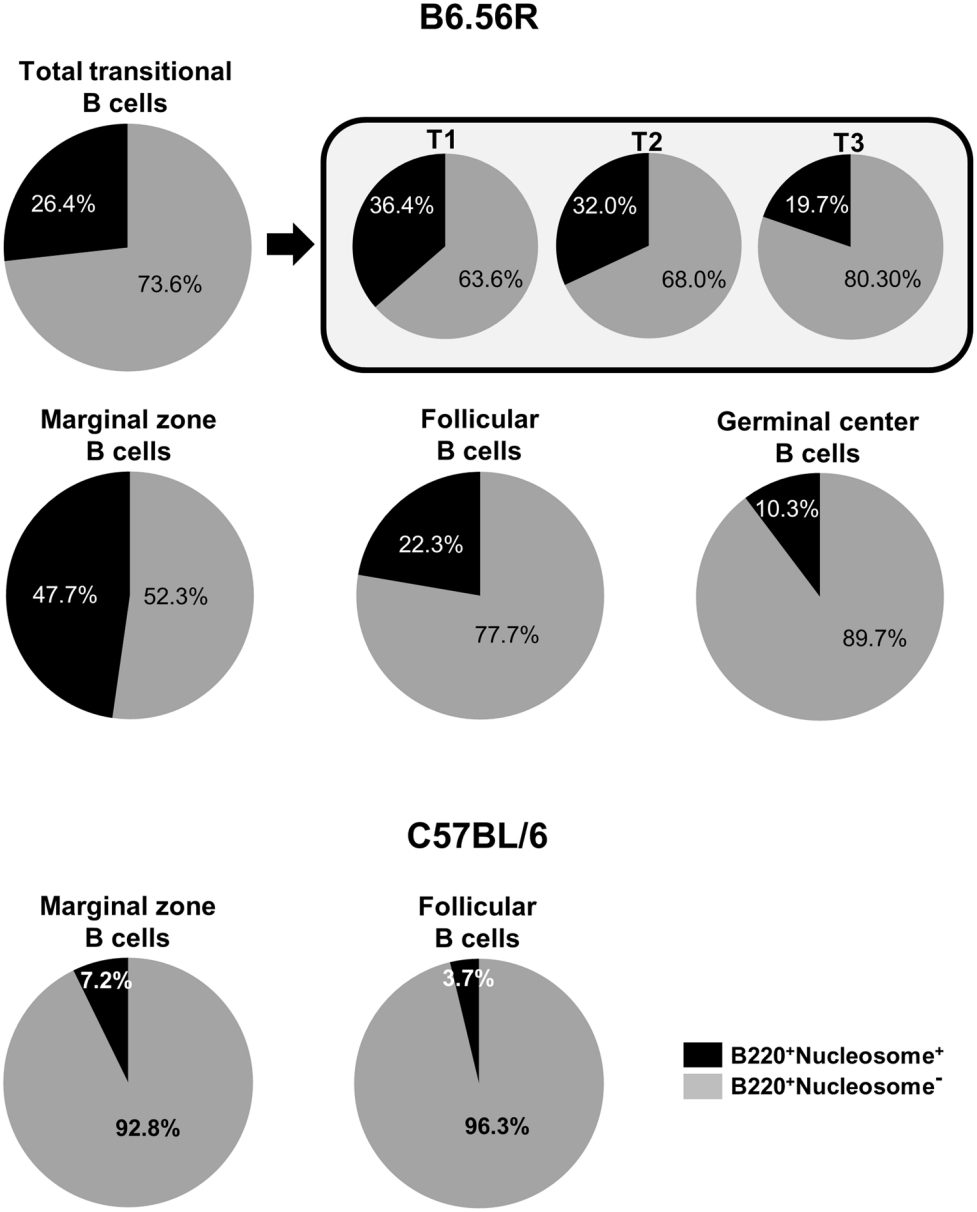
Composition of splenic B cell compartments. Repartition of nucleosome^+^ *(black)* and nucleosome^−^ (*grey*) B cell splenic subsets in B6.56R and C57BL/6 mice. n ≥ 9; spleen; mice aged from 2 to 8 months old.

Based on these results, we represented the cellular composition of each B cell subset. With respect to the transitional population in B6.56R mice, the proportion of nucleosome^+^ B cells was relatively high (26.4%) and the lowest representation of nucleosome^+^ B cells was in the T3 subset. MZ B cells had the highest cellular proportion of nucleosome^+^ B cells, as they represented almost half (47.7%) of the cellular content, whereas anti-nucleosome B cells represented 22.3% of FO B cells (Fig. 6). In the same manner, in C57BL/6 mice, MZ B cell population displayed a higher proportion of nucleosome^+^ cells compared to FO B cell compartment (Fig. 6). Finally, in B6.56R mice, GC B cells constituted the compartment with the lowest proportion of autoreactive B cells (10.3%) (Fig. 6). Altogether, these results are likely the consequence of tolerance mechanisms leading to sequestration of self-reactive B cells into the MZ, at the expense of FO and GC compartments.

## Discussion

The development of autoreactive B cells is tightly regulated, and failure in one or more of the tolerance mechanisms underlying elimination of autoreactive B cells may lead to autoantibody-mediated diseases. Anti-dsDNA is one of the most common autoantibodies in SLE^33^, and B6.56R mice possess self-reactive B cells producing such autoantibodies^26^.

Herein we show that B6.56R mice display an increased MZ proportion. This expansion has been directly implicated in SLE pathogenesis in some murine models^34,35^, as the MZ is a major source of autoreactive B cells^36^. It has been suggested that a chronic BCR stimulation could promote homing of B cells into the MZ^37^. In agreement with this point, we show that nucleosome^+^ B cells, recognizing an ubiquitous autoantigen, adopt a MZ phenotype. Because MZ B cells are not favored for affinity maturation or class switching^37^, nucleosome^+^ B cells in the B6.56R model could be preferentially localized in MZ to reduce the propensity of inappropriate activation. The presence of DNA reactive BCRs is likely to promote autoreactive B cells into microenvironment where the risk of any autoimmune response is decreased. However paradoxically, in the MZ B cells are more exposed to polyclonal B cell stimuli, such as lipopolysaccharides (LPS)^38,39^, suggesting additional mechanisms to prevent activation of autoreactive B cells in this location. Similarly to what was described in 56R mice on a BALB/c background, these B cells could undergo apoptosis followed by a rapid clearance by phagocytes^40^. The lower representation of self-reactive B cells within the FO B cell compartment reduces the risk of inappropriate activation, since B cells excluded from this compartment cannot recirculate through the body and have a short half-life^41–43^.

In addition, we demonstrate that nucleosome^+^ B cells fail to acquire a GC phenotype, a tolerance mechanism that has been shown to be defective in SLE patients^44^. However, B6.56R mice spontaneously produce anti-dsDNA autoAbs of both IgM and IgG isotypes^26^, suggesting that nucleosome^+^ B cells could differentiate and undergo class-switching via an extrafollicular pathway^45^.

Emigrant B cells that have passed the BM tolerance checkpoints migrate into the spleen to complete their maturation. These cells are known as transitional B cells, and can be further divided into three subsets – T1 (CD93^+^IgM^high^CD23^−^), T2 (CD93^+^IgM^high^CD23^low^) and T3 (CD93^+^IgM^low^CD23^high^)^46,47^ –. T1 B cells give rise to T2 B cells, that become FO precursors or can differentiate into T3 B cells^46–49^. The T1 stage appears to be an important tolerance checkpoint, as suggested by studies showing that BCR crosslinking induces excessive T1 B-cell apoptosis^50–52^. Further maturation of T1 B cells is marked by a BAFF-mediated survival, with an upregulation of BAFF-R^53^, and by enhancement of deletional tolerance, with an increased sensitivity to anti-IgM-mediated apoptosis^54^. Thus, self-reactive T2 B cells are usually deleted, or may be oriented towards the T3 population that remains anergic in response to BCR cross-linking^55,56^. Lupus-prone mice are characterized by a decrease in T3 B cells^47^, suggesting that this subset has an important role in autoimmunity development. Consistent with this hypothesis, we observed that this subset is more enriched in nucleosome^−^ rather than in nucleosome^+^ B cells. Thus, it seems that the T3 compartment is not a reservoir for functional autoreactive B cells but rather consists of inactive B cells that have successfully undergone tolerance. It is tempting to speculate that the T3 compartment is reduced in lupus-prone mice due to a defective tolerance process. On the contrary, the higher frequency of total T3 B cells representation in B6.56R mice could be the consequence of effective tolerance mechanisms leading to anergic autoreactive B cells unable to bind the nucleosome, explaining the lower proportion of T3 nucleosome^+^ B cells in B6.56R mice.

The suppression of autoreactivity is completed in part by receptor editing, allowing for the replacement of variable segments expressed by B cells^19,57–60^. This mechanism notably involves rearrangements of κ and eventually λ chains^61–63^. In B6.56R mice, the majority of anti-nucleosome B cells express a κ light chain, and only few express λ chains. The absence of nucleosome^+^ B cells in the putative Vλx^+^ B-cell population – i.e. κ/λ double negative cells after staining with commercially available antibodies – supports the fact that the Vλx editor is present and able to eliminate DNA binding capacity of the 56R transgene^31,32^. In addition, some κ^+^/λ^+^ B cells, that are known to be located in the MZ^64^, are present. These cells have undergone several rounds of receptor editing in a timeframe that is long enough to allow for the co-rearrangement of a λ and new κ chain. This isotypic inclusion leads to the expression of a λ chain that allows DNA binding by the 56R transgene and one κ that abrogates this interaction^64^. It was suggested that this dual expression leads to a strong decrease of autoreactive BCR density, and such partial autoreactive B cells only display a low affinity towards the autoantigen^64^. Our results confirm this hypothesis with an absence of nucleosome binding among dual receptor expressing B cells, showing that this population is unable to interact with labeled nucleosomes because of diluted autoreactive BCR reactivity. However, as proposed by Li *et al*., the low residual autoreactivity could be sufficient to explain the homing of these B cells into the MZ^31,64^, where their sequestration may prevent their GC entry to control their pathogenic properties.

Taken together, these data directly highlight the existence of peripheral tolerance mechanisms that limit the development of autoreactive FO B cells and that render them unable to acquire a GC phenotype. This process results in an accumulation of self-reactive MZ B cells. Our flow cytometry assay, using labeled nucleosomes, can directly and quickly elicit the phenotype of these autoreactive B cells and give information about their preferential location. It is a useful tool for the study of B cell tolerance breakdown mechanisms, for example in B6.56R crossed with autoimmune prone models. This could provide new clues to elucidate the pathogenesis of autoimmune diseases, such as SLE.

## Materials and Methods

### Mice

All experiments were carried out in accordance with the European Community guidelines on the protection of animals used for scientific purposes (Directive 2010/63/UE) and approved by the Regional Ethics Committee of Strasbourg (CREMEAS). 2 to 8 months-old C57BL/6 or B6.56R mice were bred in our animal facility (approved by French Veterinary Services, #F67–482–2). B6.56R transgenic mice were genotyped by PCR amplification of tail DNA^65^. 56R mice on C57BL/6 background were kindly provided by M. Weigert (Department of Pathology, University of Chicago, Chicago, IL, USA).

### Nucleosome isolation, labeling and characterization

Labeled nucleosome were produced as previously described^22^. Briefly, nucleosomes were isolated from L1210 cells and DNA concentration was measured (dilution 1:100 in 0.1% (v/v) SDS solution). 200 µL of unlabeled nucleosome solution was mixed with 250 µL of buffer (10 mM Tris-HCl, 0.7 mM EDTA, 3.6 M NaCl). 5-fold molar excess of AlexaFluor488 C_5_-maleimide (Molecular Probes) was added and incubated 3 hours at room temperature. Successive dialyses against solutions of decreasing ionic force were performed. DNA concentration was measured (dilution 1:100 in 0.1% (v/v) SDS solution) and the samples were stored at −20 °C.

### Flow Cytometry

Flow cytometry was performed as previously described^22^. All steps are done in PBE (PBS (137 mM NaCl, 2.7 mM KCl, 10 mM Na_2_HPO_4_, 2 mM KH_2_PO_4_), 0.5% (w/v) BSA, 2 mM EDTA) buffer on ice, unless otherwise specified. Briefly, 1*10^6^ splenocytes were incubated with 100 µL of nucleosome solution (2 µg of DNA/mL) and incubated 20 min at 4 °C. The cells were washed twice in PBE/0.1% (v/v) Tween 20 and classical surface staining was performed for 15 min at 4 °C. Cell viability was assessed by incubation with Fixable Viability Dye eFluor 780 (eBioscience) following the manufacturer’s protocol. Acquisition was performed on a Gallios flow cytometer (Beckman Coulter). Data analysis was performed using Kaluza software (Beckman Coulter) with “logicle” compensation^66^. A comprehensive list of anti-mouse antibodies used for this experiment includes: IgM^a^ (clone DS-1), IgM^b^ (clone AF6–78), B220 (clone RA3–6B2), CD95 (clone Jo2), kappa (187.1) and lambda 1, 2 and 3 (R26–46) from BD Biosciences; CD23 (clone B3B4) and CD93 (clone AA4.1) from eBioscience; CD138 (clone 281–2) and GL7 (clone GL7) from BioLegend; streptavidin-AlexaFluor700 from Molecular Probes.

### Statistics

Statistical comparison between groups was carried out using nonparametric two-tailed Wilcoxon matched paired signed-rank test or nonparametric two-tailed Mann-Whitney U-test using Prism 5.0 (GraphPad Software Inc.). All data were presented as mean ± standard error of the mean (SEM). A p-value less than 0.05 was considered as significant.

## Electronic supplementary material

Supplementary information
