# Supplementary material for: Phenotyping of autoreactive B cells with labeled nucleosomes in 56R transgenic mice
Source: Sci Rep. 2017 Oct 16;7:13232. doi: 10.1038/s41598-017-13422-z (PMC5643551; doi:10.1038/s41598-017-13422-z)
Supplement: Supplementary file 1 — Supplementary information [file 41598_2017_13422_MOESM1_ESM.doc]

**SUPPLEMENTARY INFORMATION**

**Scientific Reports**

**Phenotyping of autoreactive B cells with labeled nucleosomes in 56R transgenic mice**

Vincent Gies,^1,2^ Delphine Bouis,^1*^ Mickael Martin,^1,2*^ Jean-Louis Pasquali,^1,2,3^ Thierry Martin,^1,2,3^ Anne-S. Korganow,^1,2,3^ Pauline Soulas-Sprauel,^1,2,4**^

^1^CNRS UPR 3572 "Immunopathology and Therapeutic Chemistry"/Laboratory of Excellence Medalis, Institute of Molecular and Cellular Biology (IBMC), Strasbourg, France.

^2^Department of Clinical Immunology and Internal Medicine, National Reference Center for Autoimmune Diseases, Hôpitaux Universitaires de Strasbourg, Strasbourg, France.

^3^UFR Médecine, Université de Strasbourg, Strasbourg, France.

^4^UFR Sciences pharmaceutiques, Université de Strasbourg, Illkirch-Graffenstaden, France.

*These authors contributed equally to the work

****Corresponding author:**

Pauline SOULAS-SPRAUEL, PharmD, PhD

CNRS UPR 3572 “Immunopathology and Therapeutic Chemistry”

Institute of Molecular and Cellular Biology (IBMC)

15 rue René Descartes, 67084 Strasbourg Cedex, FRANCE

Telephone number: + 33 3 88 41 70 25

Fax: + 33 3 88 61 06 80

E-mail address: [pauline.soulas@ibmc-cnrs.unistra.fr](mailto:pauline.soulas@ibmc-cnrs.unistra.fr)

**SUPPLEMENTARY TABLES**

**Supplementary Table 1.** Absolute numbers of splenic B-cell subsets in C57BL/6 mice and B6.56R mice. n≥5 in each group; spleen; mean ± SEM. Two-tailed Mann-Whitney U-test. **P*<.05, ***P*<.005. Red: increased value; green: decreased value.

|  | | **C57BL/6** | **B6.56R** |
| --- | --- | --- | --- |
| Total B cells  (B220^+^) | *10^6^ | 34.4 ±5.3 | 12.4 ±2.1** |
|  | [range] | [19.8-56.3] | [6.7-29.6] |
| Transitional  (B220^+^CD93^+^IgM^low/high^CD23^-^/^low/high^) | *10^6^ | 2.1 ±0.2 | 0.5 ±0.1** |
|  | [range] | [1.3-2.6] | [0.15-1.0] |
| Marginal zone  (B220^+^CD93^-^IgM^high^CD23^-/low^) | *10^6^ | 2.9 ±0.4 | 4.8 ±0.6* |
|  | [range] | [2.3-4.5] | [2.9-5.8] |
| Follicular  (B220^+^CD93^-^IgM^low^CD23^high^) | *10^6^ | 23.8 ±3.2 | 8.1 ±2.4* |
|  | [range] | [14.2-34.0] | [3.4-17.4] |
| Germinal center  (B220^+^GL7^high^CD95^high^) | *10^6^ | 0.07 ±0.01 | 0.06 ±0.02 |
|  | [range] | [0.03-0.10] | [0.04-0.12] |

**Supplementary Table 2.** Main features of peripheral nucleosome^+^ B cells in BA.56R mice. n≥5; spleen; mean ± SEM.. Statistical analysis between nucleosome^+^ B cells and nucleosome^-^ B cells using two-tailed Mann-Whitney U-test (light chain) or two-tailed Wilcoxon matched paired signed-rank test (Phenotype). **P*<.05, ***P*<.005, ***P*<.001. Red: increased value; green: decreased value

|  | | **Total B cells**  **(% of B220+)** | **Nucleosome^-^ B cells**  **(% of B220+Nucleosome^-^)** | **Nucleosome^+^ B cells**  **(% of B220+Nucleosome^+^)** |
| --- | --- | --- | --- | --- |
| **LIGHT CHAIN** | Κ | 77.8 ±1.4 | 73.9 ±1.3 | 92.6 ±0.8** |
|  | λ | 2.6 ±0.2 | 2.8 ±0.2 | 2.1 ±0.2* |
|  | Κ^+^/ λ^+^ | 5.2% ±0.8 | 6.2 ±1.0 | 3.5 ±0.5* |
|  | Κ^-^/ λ^-^ | 14.4 ±1.0 | 17.8 ±1.2 | 1.9 ±0.2** |
| **PHENOTYPE** | Transitional | 2.1 ±0.3 | 2.3 ±0.3 | 1.8 ±0.2* |
|  | *T1* | *0.48 ±0.08* | *0.44 ±0.08* | *0.56 ±0.10* |
|  | *T2* | *0.45 ±0.06* | *0.45 ±0.06* | *0.47 ±0.08* |
|  | *T3* | *1.17 ±0.19* | *1.36 ±0.23* | *0.72 ±0.10*** |
|  | Marginal zone | 36.2 ±2.0 | 27.2 ±1.7 | 56.7 ±2.7*** |
|  | Follicular | 48.2 ±1.6 | 54.1 ±1.5 | 35.0 ±1.9*** |
|  | Germinal center | 0.39±0.06 | 0.58 ±0.09 | 0.14 ±0.02** |

**SUPPLEMENTARY FIGURES**

**Supplementary Figure 1.** Labeled nucleosomes selectively stained a subset of B cells carrying the 56R transgenic heavy chain in B6.56R mice. (**a**) Representative plots of nucleosome staining in B6.56R and C57BL/6 mice. (**b)** Representative plots of nucleosome^+^ B cells among IgM^a+^ cells or IgM^b+^ B cells in B6.56R.

**Supplementary Figure 2.** Discrepancy between nucleosome^+^ or nucleosome^-^ IgM^a+^ B cells. Frequency of total B220^+^ B cells (●), nucleosome^-^ B cells (■) and nucleosome^+^ B cells (▲) in MZ, FO, and GC B cell compartments (**a**) and in transitional compartment, including T1, T2 and T3 (**b**) in B6.56R mice (indicated as percentages among B220^+^IgM^a+^, B220^+^IgM^a+^nucleosome^-^ or B220^+^IgM^a+^nucleosome^+^ cells). n=14; spleen; mice aged from 2 to 8 months old. Two-tailed Wilcoxon matched paired signed-rank test. **P*<.05, ** *P*<.005, ****P*<.001.
